# Morphological and molecular characteristics of *Malayfilaria sofiani* Uni, Mat Udin & Takaoka n. g., n. sp. (Nematoda: Filarioidea) from the common treeshrew *Tupaia glis* Diard & Duvaucel (Mammalia: Scandentia) in Peninsular Malaysia

**DOI:** 10.1186/s13071-017-2105-9

**Published:** 2017-04-20

**Authors:** Shigehiko Uni, Ahmad Syihan Mat Udin, Takeshi Agatsuma, Weerachai Saijuntha, Kerstin Junker, Rosli Ramli, Hasmahzaiti Omar, Yvonne Ai-Lian Lim, Sinnadurai Sivanandam, Emilie Lefoulon, Coralie Martin, Daicus Martin Belabut, Saharul Kasim, Muhammad Rasul Abdullah Halim, Nur Afiqah Zainuri, Subha Bhassu, Masako Fukuda, Makoto Matsubayashi, Masashi Harada, Van Lun Low, Chee Dhang Chen, Narifumi Suganuma, Rosli Hashim, Hiroyuki Takaoka, Mohd Sofian Azirun

**Affiliations:** 1 Institute of Biological Sciences, Faculty of Science, University of Malaya, Kuala Lumpur, 50603 Malaysia; 2 Department of Parasitology, Graduate School of Medicine, Osaka City University, Osaka, 545-8585 Japan; 3 Department of Environmental Medicine, Kochi Medical School, Kochi University, Nankoku, 783-8505 Japan; 4 Walai Rukhavej Botanical Research Institute, Mahasarakham University, Mahasarakham, 44150 Thailand; 5 ARC-Onderstepoort Veterinary Institute, Private Bag X05, Onderstepoort, 0110 South Africa; 6 Department of Parasitology, Faculty of Medicine, University of Malaya, Kuala Lumpur, 50603 Malaysia; 7 UMR7245, MCAM, Muséum National d’Histoire Naturelle, Paris, 75005 France; 8 Research Promotion Institute, Oita University, Oita, 879-5593 Japan; 9 Department of International Prevention of Epidemics, Division of Veterinary Science, Graduate School of Life and Environmental Sciences, Osaka Prefecture University, Osaka, 598-8531 Japan; 10 Laboratory Animal Center, Graduate School of Medicine, Osaka City University, Osaka, 545-8585 Japan; 11 Tropical Infectious Diseases Research & Education Centre, University of Malaya, Kuala Lumpur, 50603 Malaysia

**Keywords:** Filarial nematodes, Lymphatic filariosis, Molecular identification, Onchocercidae, Scandentia, Southeast Asia

## Abstract

**Background:**

The filarial nematodes *Wuchereria bancrofti* (Cobbold, 1877), *Brugia malayi* (Brug, 1927) and *B. timori* Partono, Purnomo, Dennis, Atmosoedjono, Oemijati & Cross, 1977 cause lymphatic diseases in humans in the tropics, while *B. pahangi* (Buckley & Edeson, 1956) infects carnivores and causes zoonotic diseases in humans in Malaysia. *Wuchereria bancrofti*, *W. kalimantani* Palmieri, Pulnomo, Dennis & Marwoto, 1980 and six out of ten *Brugia* spp. have been described from Australia, Southeast Asia, Sri Lanka and India. However, the origin and evolution of the species in the *Wuchereria-Brugia* clade remain unclear. While investigating the diversity of filarial parasites in Malaysia, we discovered an undescribed species in the common treeshrew *Tupaia glis* Diard & Duvaucel (Mammalia: Scandentia).

**Methods:**

We examined 81 common treeshrews from 14 areas in nine states and the Federal Territory of Peninsular Malaysia for filarial parasites. Once any filariae that were found had been isolated, we examined their morphological characteristics and determined the partial sequences of their mitochondrial cytochrome *c* oxidase subunit 1 (*cox*1) and 12S rRNA genes. Polymerase chain reaction (PCR) products of the internal transcribed spacer 1 (ITS1) region were then cloned into the pGEM-T vector, and the recombinant plasmids were used as templates for sequencing.

**Results:**

*Malayfilaria sofiani* Uni, Mat Udin & Takaoka, n. g., n. sp. is described based on the morphological characteristics of adults and microfilariae found in common treeshrews from Jeram Pasu, Kelantan, Malaysia. The Kimura 2-parameter distance between the *cox*1 gene sequences of the new species and *W. bancrofti* was 11.8%. Based on the three gene sequences, the new species forms a monophyletic clade with *W. bancrofti* and *Brugia* spp. The adult parasites were found in tissues surrounding the lymph nodes of the neck of common treeshrews.

**Conclusions:**

The newly described species appears most closely related to *Wuchereria* spp. and *Brugia* spp., but differs from these in several morphological characteristics. Molecular analyses based on the *cox*1 and 12S rRNA genes and the ITS1 region indicated that this species differs from both *W. bancrofti* and *Brugia* spp. at the genus level. We thus propose a new genus, *Malayfilaria*, along with the new species *M. sofiani*.

**Electronic supplementary material:**

The online version of this article (doi:10.1186/s13071-017-2105-9) contains supplementary material, which is available to authorized users.

## Background

Filarial nematodes (superfamily Filarioidea Weinland, 1858) parasitise all classes of vertebrates other than fishes and are transmitted by haematophagous arthropods [1–3]. *Wuchereria bancrofti* (Cobbold, 1877), *Brugia malayi* (Brug, 1927) and *B. timori* Partono, Purnomo, Dennis, Atmosoedjono, Oemijati & Cross, 1977 parasitise humans and are the causative agents of human lymphatic diseases [4–9]. These parasites not only lead to direct health problems but add to the causative agents of tropical diseases which create public health burdens that hinder socioeconomic development in tropical and subtropical regions of the world [10–12].

Recently, there has been a worldwide increase in the incidence of zoonotic infections in humans caused by filarial nematodes that parasitise other animals, such as dogs, cats, cattle and wild boars. It is believed that global warming, deforestation and human demographics are affecting the transmission of parasites by bridging the gaps between vectors, host animals and humans [13]. For example, the number of cases of zoonotic onchocercosis has increased in Europe, the USA and Japan, with infections expanding into areas where the diseases had not previously been reported [14–16]. In Malaysia, *B. pahangi* (Buckley & Edeson, 1956), a parasite of cats, dogs and wild carnivores, has recently been found to cause zoonotic diseases in humans [17, 18].

A total of 34 species of filarioids from 21 genera have previously been recorded from vertebrates in Malaysia [3, 19]. Species in the genera *Wuchereria* Silva Araujo, 1877 and *Brugia* Buckley, 1960 have biological similarities with regard to the vectors used and their location in the definitive hosts [4]. Recent molecular analyses have indicated that they are closely related, forming a single clade [20–26]. Based on their geographical distribution, Bain [27] suggested that *W. bancrofti* in humans spread from the Oriental Pacific area through the tropical belt and that *Wuchereria* and *Brugia* may have diversified in Southeast Asia. However, the origin and evolution of these species remain to be clarified [23].

To further elucidate the diversity and phylogeny of filarial parasites in Malaysia, we searched for parasites in several wild animal species, including common treeshrews. Common treeshrews (*Tupaia glis* Diard & Duvaucel) (Mammalia: Scandentia) are small-bodied mammals that are distributed throughout southern Thailand, Peninsular Malaysia and Borneo. In Peninsular Malaysia, they are still commonly seen in agricultural areas and human habitats. Treeshrews belong to the order Scandentia, which includes the family Tupaiidae [28–30]. Two filarial parasites, i.e. *Brugia tupaiae* Orihel, 1966 and *Mansonella* (*Tupainema*) *dunni* (Mullin & Orihel, 1972) have previously been described from common treeshrews in Malaysia [31–34].

During this investigation, we found filarial nematodes in common treeshrews captured in Kelantan, on the east coast of Peninsular Malaysia. None of the specimens collected match any of the hitherto described species or genera of filariae [2, 3, 19]. A new genus and species are, therefore, proposed to accommodate the present nematodes, which are similar to representatives of *Wuchereria* and *Brugia*, but distinct from them on the basis of several morphological characters. Analyses of the cytochrome *c* oxidase subunit 1 (*cox*1) and 12S rRNA genes and the internal transcribed spacer 1 (ITS1) region supported our findings that the new species differs from *W. bancrofti* and *Brugia* spp. at the genus level, and phylogenetic tree topologies depicted it as sister species of the *Wuchereria-Brugia* clade.

## Methods

### Collection of hosts and parasite material

Between June 2012 and March 2016, we captured 81 common treeshrews from primary and secondary (second-growth) forests in 14 areas in nine states and the Federal Territory of Peninsular Malaysia: 4 animals from Bukit Kanthan, Perak; 3 from Ulu Kenas, Perak; 18 from Jeram Pasu, Kelantan; 4 from Jeram Linang, Kelantan; 9 from Taman Negara Kelantan; 3 from Genting Highlands, Pahang; 5 from Tioman Island, Pahang; 11 from Langkawi Island, Kedah; 7 from Krubong, Melaka; 2 from Perlis State Park; 5 from Gunung Belumut Forest Reserve, Johor; 1 from Kampung Pantai Batu Bukit, Terengganu; 4 from Ulu Gombak, Selangor and 5 from Kuala Lumpur, the Federal Territory. All animals were captured with permission of the Department of Wildlife, Malaysia, using box cage traps baited with palm oil kernels or bananas. The common treeshrews were anaesthetised and sacrificed in accordance with the policy and protocols approved by the Animal Care Committee, University of Malaya, Kuala Lumpur, Malaysia. To obtain adult filariae, we dissected the lymphatic tissues, peritoneal cavity and subcutaneous connective tissues of common treeshrews under a stereomicroscope.

### Morphological methods

#### Newly collected material

Thick blood smears were made and stained with 3% Giemsa solution (pH 7.4), and skin snips were taken from the face, ears, back, abdomen and tail, as described by Uni et al. [35]. We then searched for microfilariae in the blood smears and skin snips under a compound microscope. We also recorded the length and width of microfilariae taken from the uteri of adult worms.

Isolated adult worms were then transferred into either 70% ethanol for the examination of fixed worms from one common treeshrew (group A) or into phosphate-buffered saline (pH 7.4) for the examination of dead unfixed worms from another individual (group B). The fixed adult worms in group A were cleared in lactophenol solution (R & M Chemicals, Essex, UK) and drawn using a compound microscope equipped with a camera lucida (Olympus U-DA, Olympus, Tokyo, Japan). The midbody region of a fixed female was embedded in paraffin and sections were stained with haematoxylin and eosin. Dead worms that had neither been fixed nor cleared (group B) were also drawn using the same apparatus. For each worm in groups A and B, we recorded body length, body width, distance between anterior end and nerve ring, distance between anterior end and vulva, length of oesophagus, spicules and tail. Metrical data are presented as the range and measurements are in micrometres unless otherwise indicated.

#### Additional material examined

We also examined specimens of *B. malayi* (taken from a human in Pahang, Malaysia, in 1999) and *B. pahangi* (taken from a domestic cat at Kampung Kerinchi, Kuala Lumpur, in 1999), archived at the Department of Parasitology, Faculty of Medicine, University of Malaya, Malaysia, for comparative purposes. One female (ID no. M1) and one male (ID no. M2) of *B. malayi* and one female (ID no. M3) and one male (ID no. M4) of *B. pahangi* were fixed in hot 70% methanol and immersed in glycerine. Microfilariae of each species were examined on thick-blood-film slides stained with Giemsa solution (ID no. MS1 for *B. malayi* and ID no. MS2 for *B. pahangi*).

### Molecular methods

Three females (F0, F1 and F2) were transferred directly into 80% ethanol. DNA extraction, PCR amplification and sequencing were performed as described previously [36–38] to determine the partial sequences of the mitochondrial *cox*1 and 12S rRNA genes. We also cloned the PCR products of the ITS1 region into pGEM-T vectors and determined the sequences of the recombinant plasmid [39]. *Filaria martis* Gmelin, 1790 was selected as the outgroup for *cox*1 and 12S rRNA gene analyses, and *Onchocerca* spp. were selected for analyses based on ITS1. Sequence data were deposited in the GenBank database (see taxonomic summary). The corresponding GenBank accession numbers of other species used to compare the present specimens and to determine their phylogenetic relationships, are provided in the figures. We used the Kimura 2-parameter model [40] to estimate evolutionary distances between species and calculated K2P distances between species of filarial parasites in MEGA6 as an estimate of the accumulated number of nucleotide substitutions per site [41].

Phylogenetic trees of the nucleotide sequences of the genes were constructed using neighbour-joining (NJ) and maximum-likelihood (ML) methods [41, 42]. Analyses were performed based on 569 bp of the *cox*1 gene, 319 bp of the 12S rRNA gene and 495 bp of the ITS1 region using MEGA6. Analyses were also performed based on the concatenated alignment (*cox*1 + 12S rDNA, 888 bp).

## Results

**Class Secernentea von Linstow, 1905**

**Family Onchocercidae Leiper, 1911**

**Subfamily Onchocercinae Leiper, 1911**

### *Malayfilaria* Uni, Mat Udin & Takaoka n. g.

#### Diagnosis

Anterior extremity slightly expanded into cephalic bulb not set-off from body. Head papillae comprising four labial and four cephalic papillae. Buccal cavity narrow, with pre-oesophageal cuticular ring. Nerve-ring situated at level of muscular oesophagus. Oesophagus divided into short muscular and long glandular parts. Salient cuticular annules present in region of midbody. *Female*. Vulva at level of anterior part of glandular region of oesophagus. Vagina with two bends. Ovejector long, muscular. Uteri didelphic and opisthodelphic. Tail extremity with two small ventrolateral lappets. *Male.* Area rugosa precloacal. Caudal alae absent. Spicules unequal in length and dissimilar in shape; left spicule long and slender, divided into handle and simple blade, right spicule short and robust. Gubernaculum present. Caudal papillae and two small lappets on tail end present. Tail long, its length almost twice (1.9×) width of body at anus. Microfilariae sheathed, with one terminal nucleus; present in peripheral blood. *Type-species*: *Malayfilaria sofiani* Uni, Mat Udin & Takaoka n. sp. *Etymology*: The generic name is derived from Malaya, the former name of Malaysia.

### *Malayfilaria sofiani* Uni, Mat Udin & Takaoka n. g., n. sp.

***Type-host*** **:** *Tupaia glis* Diard & Duvaucel (Scandentia: Tupaiidae), common treeshrew.

***Type-locality*** **:** Jeram Pasu (5.815454, 102.348309), Kelantan, Malaysia.

***Type-material*** **:** The holotype female (MNHN 95YT) and the allotype male (MNHN 96YT) were deposited in the Muséum National d’Histoire Naturelle, Paris, France. Paratypes (six females: B2, P2 and KE2–5 and nine males: B1, P1, P3–5 and KEM2–5) were deposited in the Institute of Biological Sciences, University of Malaya, Malaysia (accession numbers: Ms-B2, Ms-P2, Ms-KE2–5, Ms-B1, Ms-P1 and Ms-KEM2–5). Collection dates: 16.xi.2013 (group A in treeshrew ID no. 3) and 25.iii.2016 (group B with type specimens in treeshrew ID no. 6).

***Site in host*** **:** Adult worms invade the tissues surrounding the lymph nodes of the neck of treeshrews. Microfilariae circulate in the blood of treeshrews.

***Prevalence and intensity of infection*** **:** Two of 81 (2.5%) treeshrews examined were infected with 12 adult worms each: six female and six male worms in tree shrew ID no. 3, and seven female and five male worms in treeshrew ID no. 6. Microfilariae were present in five of the examined hosts (6.2%).

***Representative DNA sequences*** **:** Sequence data were deposited in the GenBank database as follows: *cox*1 (KX944563–KX944565); 12S rRNA (KX944560–KX944562); and ITS1 (KX944548–KX944559).

***ZooBank registration*** **:** To comply with the regulations set out in article 8.5 of the amended 2012 version of the *International Code of Zoological Nomenclature* (ICZN) [43], details of the new species have been submitted to ZooBank. The Life Science Identifier (LSID) of the article is urn:lsid:zoobank.org:pub:36584586-131A-4C30-A529-68C47E5920E6. The LSID for the new name *Malayfilaria* n. g. is urn:lsid:zoobank.org:act:CE9E406C-BD20-4BD6-802C-B00FABCF8527 and the LSID for the new name *Malayfilaria sofiani* Uni, Mat Udin & Takaoka n. sp. is urn:lsid:zoobank.org:act:22CC40B9-2427-414D-9D74-0292B7BF53D1.

***Etymology*** **:** The specific name is chosen in honour of Professor Dr Mohd Sofian Azirun, former Dean of the Faculty of Science, University of Malaya, for his contributions to entomology and parasitology in Malaysia.

### Description

**Fig. 1:**
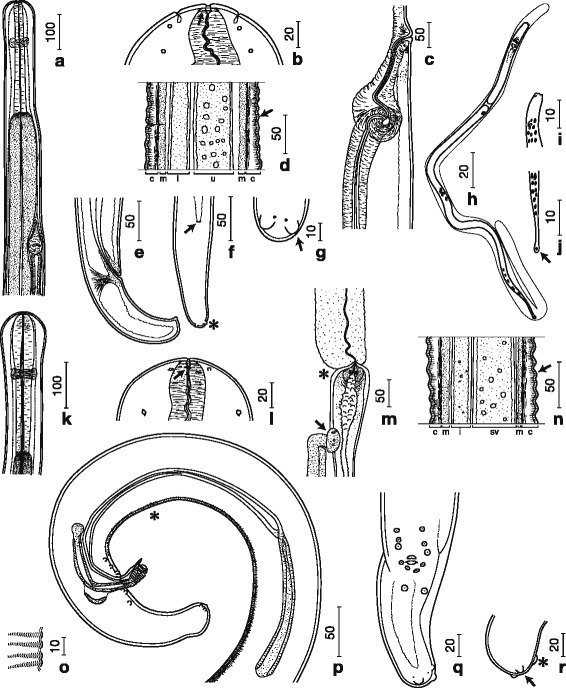
Camera lucida drawings of *Malayfilaria sofiani* n. g., n. sp. Females (**a**–**g**), microfilariae (**h**–**j**) and males (**k**–**r**). **a** Anterior part, right lateral view. **b** Head, dorsoventral view, showing pre-oesophageal cuticular ring (arrow). **c** Vagina, right lateral view; **d** Annules (arrow) at midbody; *Abbreviations*: c, cuticle; m, muscle; i, intestine; u, uterus. **e** Posterior part, right lateral view. **f** Posterior part, ventral view, showing anus (arrow) and lappets (*). **g** Lappets (arrow) with phasmidial pore at posterior end, ventral view. **h** Microfilaria with sheath. **i** Head, dorsoventral view. **j** Tail tip with terminal nucleus (arrow). **k** Anterior part, lateral view. **l** Head with amphid (arrow), lateral view. **m** Oesophago-intestinal junction (*) and apex of testis (arrow). **n** Annules (arrow) at midbody; *Abbreviations*: c, cuticle; m, muscle; i, intestine; sv, seminal vesicle. **o** Area rugosa, lateral view. **p** Posterior part, right lateral view, showing area rugosa (*). **q** Posterior part, ventral view. **r** Tail tip with knob (*) and lappets (arrow), lateral view. *Scale-bars* are in micrometres

***General.*** Adult worms small, slender, tapering toward both ends. Anterior extremity slightly dilated, not set-off from remainder of body (Fig. 1a). Labial and cephalic papillae arranged in a circle of four each (Fig. 1b). Amphids lateral, on level of labial papillae. Mouth opening small, followed by pre-oesophageal cuticular ring. Nerve ring surrounding oesophagus on level of muscular part. Deirids not observed. Oesophagus clearly divided into short muscular and long glandular parts. Intestine narrower than glandular oesophagus. Cuticle in midbody region with distinct annules comprising several transverse striations (Fig. 1d); in transverse section, cuticle thickened at lateral chords. Tail moderately long in both sexes.

**Fig. 2:**
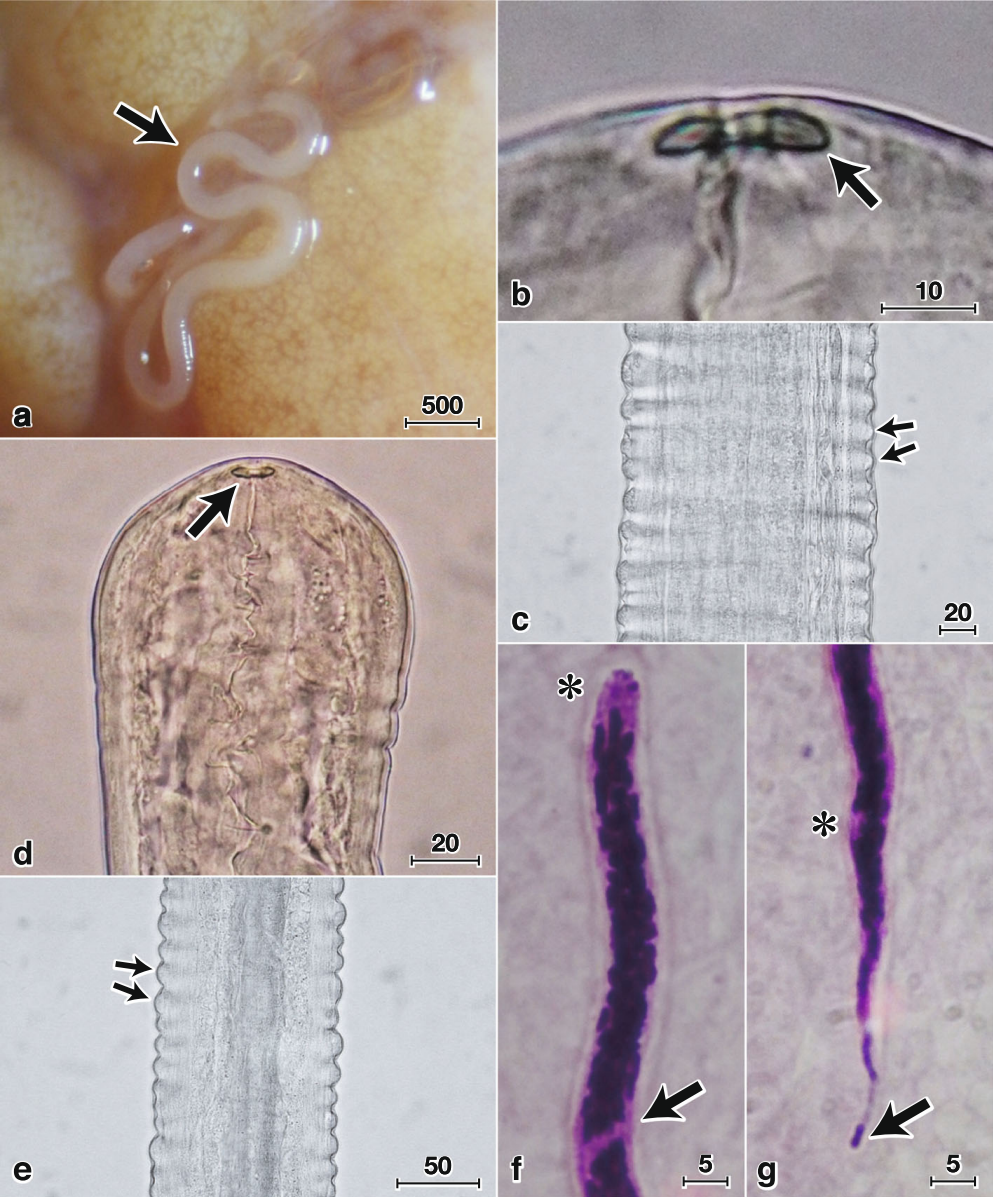
Micrographs of *Malayfilaria sofiani* n. g., n. sp. Females (**a**–**c**), males (**d**–**e**) and microfilariae (**f**–**g**). **a** Adult female (arrow) in tissue surrounding the lymph nodes of the neck of a treeshrew (*Tupaia glis*). **b** Pre-oesophageal cuticular ring (arrow). **c** Annules (arrows) at midbody region. **d** Bulbous head with pre-oesophageal cuticular ring (arrow). **e** Annules (arrows) at midbody region. **f** Anterior part with cephalic space (*) and nerve ring (arrow) (Giemsa staining). **g** Posterior part with anal pore (*) and terminal nucleus (arrow) (Giemsa staining). *Scale-bars* are in micrometres

***Female.*** [Based on the holotype (MNHN 95YT) and nine complete and four fragmented specimens; Figs. 1a–g, 2a–c; Additional file 1: Table S1.] Head bulbous, 113–128 wide, 111–125 long (Fig. 1a). Buccal cavity with pre-oesophageal cuticular ring 18–19 wide, 3 high (Fig. 1b). Posterior part of oesophagus very long. Vulva a transverse slit at level of anterior part of glandular oesophagus. Vagina, 219 long, 78 wide, with an initial transverse part, followed by a slight bend and a longer posteriorly directed straight part, followed by a second, sharper bend prior to junction with muscular ovejector (Fig. 1c). Ovejector straight, parallel to oesophagus and posteriorly directed. Tail bent ventrally, extremity rounded, with a pair of rounded ventrolateral lappets (Fig. 1f–g). Phasmids situated at base of lappets (Fig. 1g).

***Male.*** [Based on the allotype (MNHN 96YT) and nine complete and two fragmented specimens; Figs. 1k–r, 2d–e; Additional file 1: Table S1.] Head bulbous, 109 wide, 103–113 long (Figs. 1k, 2d). Buccal cavity with pre-oesophageal cuticular ring 13–15 wide, 4 high (Figs. 1l, 2d). Oesophagus consisting of muscular and long glandular parts (Fig. 1k). Apex of testis at 6.4 mm from anterior extremity, slightly posterior to oesophago-intestinal junction (Fig. 1m). Area rugosa precloacal, consisting of 181–443 raised transverse bands of short longitudinal cuticular crests, 2 high and 4–6 apart, located at 123–863 from tail end (Fig. 1o–p). Left spicule simple, divided into thick-walled handle and approximately twice longer blade with pointed tip. Right spicule divided into thick-walled proximal part and thinner-walled distal part, with distal bulb encircled by 8–9 transverse ridges (Fig. 1p). Spicule ratio 3.1–3.8:1. Gubernaculum crescent-shaped, 3 wide in lateral view (Fig. 1p); horseshoe-shaped, 43 long and 3 wide in median view. Caudal alae absent. Caudal papillae comprise unpaired ventral median papilla anterior to cloaca and six pairs of subventral papillae arranged as follows: pairs 1–3 precloacal, pair 4 adcloacal, pair 5 postcloacal, slightly more ventrally oriented, pair 6 slightly larger, at level of anterior fourth of tail. Tail bent ventrally, its extremity slightly bulbous, with single large subterminal knob, slightly shifted to left side; single pair of rounded ventrolateral lappets present on tail tip (Fig. 1q–r). Phasmids situated at base of lappets.

***Microfilaria.*** [Figs. 1h–j, 2f–g.] Sheathed unfixed microfilariae (*n* = 10) from uteri of a filarial worm 205–245 long and 5 wide (Additional file 1: Table S1, group B). Sheathed microfilariae (*n* = 10) in thick blood films from a common treeshrew (Additional file 1: Table S1, group A) 183–240 long and 5–6 wide. Cephalic space 4–8 (2–4% of body length); distance between anterior end and nerve-ring 37–50 (22–27% of body length) (Figs. 1h, 2f); distance between anterior end and excretory pore 59–70 (32–38% of body length); distance between anterior end and anal pore 143–170 (77–87% of body length) (Fig. 2g). Tail 25–43 long (13–23% of body length) with single nucleus at tail end (Figs. 1j, 2g). Small numbers of microfilariae found in skin snips of a common treeshrew (group A); sheathed, unfixed microfilariae (*n* = 10) from a skin snip 190–233 long and 5 wide.

### Prevalence and intensity

We examined 81 common treeshrews from 14 areas in nine states and the Federal Territory of Peninsular Malaysia for filarial parasites. Microfilariae of *M. sofiani* n. g., n. sp. were present in the blood of five individuals (6.2%) collected from a rubber plantation (secondary forest) in Jeram Pasu, Kelantan, Malaysia. From two of the treeshrews harbouring microfilariae, adults were recovered as well (see taxonomic summary). Only a small number of microfilariae were found in skin snips from the two hosts that harboured adult worms, whereas large numbers were found in blood smears from these two individuals. Microfilariae from both blood smears and skin snips were very similar in size. Since we also found a small number of blood cells in the skin snips, the microfilariae here appeared to have originated from the blood. We, thus, conclude that microfilariae of the new species circulate in the blood of their hosts. Microfilariae of *B. tupaiae* and *M.* (*T*.) *dunni* were not found in the blood smears examined.

### Supplementary information on *Brugia malayi* and *B. pahangi*

#### *Brugia malayi*

##### Female

[ID no. M1, Additional file 1: Table S1.] Pre-oesophageal cuticular ring 10 wide, 3 high. Head bulbous, 25 long, 38 wide, set-off from body, with neck 28 from anterior end. Annules in midbody region absent. *Male.* [ID no. M2.] Pre-oesophageal cuticular ring 8 wide, 1 high. Head bulbous, 18 long, 28 wide, set-off from body, with neck 20 from anterior end. Annules in midbody region absent; area rugosa just anterior to cloaca. *Microfilaria.* [ID no. MS1.] Tail tip of sheathed microfilariae with nucleus.

#### *Brugia pahangi*

##### Female

[ID no. M3, Additional file 1: Table S1.] Pre-oesophageal cuticular ring 8 wide, 1 high. Head bulbous, 20 long, 20 wide, set-off from body, with neck 23 from anterior end. Annules in midbody region absent. *Male.* [ID no. M4.] Pre-oesophageal cuticular ring 6 wide, 1 high. Head bulbous, 15 long, 18 wide, set-off from body, with neck 18 from anterior end. Annules in midbody region absent. *Microfilaria*. [ID no. MS2.] Tail tip of sheathed microfilariae with nucleus. This is the first study to give a detailed description of the pre-oesophageal cuticular ring in *B. pahangi*.

### Differential diagnosis

*Malayfilaria* n. g. is characterised as a member of the subfamily Onchocercinae Leiper, 1911 as defined by Anderson & Bain [2] on the basis of the following characters: an oesophagus with a well-developed glandular part, and a long tail in both sexes; vulva is located in the anterior region of the body in females, and, in males, spicules differ markedly in size and morphology, sessile caudal papillae are present, whereas caudal alae are absent.

Comparison of morphological characteristics of the present specimens with those of other onchocercine genera revealed close similarities with *Wuchereria* and *Brugia* [2, 3, 6, 7], i.e. presence of a buccal cavity with a pre-oesophageal cuticular ring, a dilated cephalic extremity, and a non-protuberant vulva as well as a rounded caudal extremity in females. Furthermore, males have a long tail, with a length almost twice the width of the body at the anus, microfilariae are sheathed and circulate in the blood. However, *Malayfilaria* n. g. can be distinguished from members of both *Wuchereria* and *Brugia* on the basis of a number of morphological characters (Additional file 1: Table S1).

*Malayfilaria* n. g. differs from *Wuchereria* as modified by Buckley [7] and characterised by Anderson & Bain [2] in having fewer caudal papillae (13 *vs c*. 24) and microfilariae with a terminal nucleus near the tail tip. In addition, the diameter of the cephalic bulb in *M. sofiani* n. g., n. sp. is 1.5 times the size of that recorded in the currently two species included in the genus *Wuchereria*, *W. bancrofti* and *W. kalimantani* Palmieri, Pulnomo, Dennis & Marwoto, 1980 [44–46], and its glandular oesophagus is six to seven times longer than that in *Wuchereria* spp. Unlike in *Wuchereria* spp., salient cuticular annules are present in the midbody region in the new species, but no cuticular bosses were found on the posterior extremity of females. While the morphology of the left spicule is similar in both *Wuchereria* spp. and *M. sofiani* n. g., n. sp. in that the blade is simple, spicules differ in length and are shorter in the new species [44, 45].

Both *Malayfilaria* n. g. and *Brugia* have microfilariae with terminal nuclei near the tail tip and a similar number of male caudal papillae (13 *vs c*.11) [2, 7]. However, *Brugia*, as defined by Buckley [7] and Anderson & Bain [2] has a left spicule with a complex blade, whereas the blade in the left spicule of *Malayfilaria* n. g. is simple. In addition, conspicuous annules in the midbody region and lappets at the posterior end, found in both sexes of *Malayfilaria* n. g., have not been recorded in *Brugia* spp. [4, 7, 8, 31, 45–54]. The glandular oesophagus in both sexes of *M. sofiani* n. g., n. sp. was seven to twelve times longer than that of all described *Brugia* spp. and the spicules of males were also longer than those of *Brugia* spp. [4, 6, 8, 31, 47–54]. When compared to *B. tupaiae*, described from the same host species, the diameter of the cephalic bulb of *M. sofiani* n. g., n. sp. was ten times that of *B. tupaiae* and the left spicule was three times longer than that of *B. tupaiae* (Additional file 1: Table S1).

In a recent comprehensive molecular phylogeny of the Onchocercidae by Lefoulon et al. [26], species of *Wuchereria* and *Brugia* formed a strongly supported clade (ONC5) consisting of two further genera of the Onchocercinae, three genera of the Dirofilariinae Sandground, 1921 and four of the Splendidofilariinae Chabaud & Choquet, 1953. Hence, we compared the morphological characteristics of *M. sofiani* n. g., n. sp. to the generic diagnoses of the respective genera as well.

Unlike *Malayfilaria sofiani* n. g., n. sp., species of *Mansonella* Faust, 1929 (Onchocercinae) do not possess a pre-oesophageal cuticular ring, the oesophagus is undivided and a gubernaculum is absent [33, 34]. In *M.* (*T.*) *dunni*, described from the same host as the new species described here, the structure of the right spicule is complex and differs from that in the present specimens [34]*.* Other than *Malayfilaria* n. g., microfilariae of species of *Breinlia* York & Maplestone, 1926 (Onchocercinae) have no sheath [2, 19, 55, 56].

In contrast with the new genus, males of species of *Loa* Stiles*,* 1905, *Pelecitus* Railliet & Henry, 1910 and *Foleyella* Seurat, 1917 (Dirofilariinae) have strongly developed caudal alae with large, pedunculate papillae [1, 2]. In addition, the oesophagus is undivided in species of both *Loa* and *Foleyella*, and species of *Pelecitus* possess well-developed lateral alae [2]. Furthermore, adults of *Loa* spp. parasitise primates [2], adults of *Pelecitus* spp. live among the tendons and muscles near the joints of the legs and feet of birds and mammals (North American lagomorphs and Australian marsupials) [1–3], and adults of *Foleyella* spp. occur in the subcutaneous connective tissues and body cavity of agamid and chamaeleonid reptiles [1, 3].

Contrary to *Malayfilaria sofiani* n. g., n. sp., species of *Cardiofilaria* Strom, 1937, *Aproctella* Cram, 1931, *Rumenfilaria* Lankester & Snider, 1982 and *Madathamugadia* Chabaud, Anderson & Brygoo, 1959 (Splendidofilariinae) have subequal spicules [2, 3, 57]. *Cardiofilaria* and *Aproctella* can be further distinguished from the new genus by caudal papillae that are arranged in a circle or semi-circle around the cloaca of males (sometimes irregularly distributed in *Cardiofilaria*), and members of both genera are found in the body cavity of birds [1, 2, 58–60]. Adult worms of *Rumenfilaria* spp. occur in the subserosal connective tissue between folds of the ruminal wall of moose, *Alces alces* (Linnaeus, 1758), in Canada and further differ from the new genus in having a smooth cuticle and nine pairs of caudal papillae [3]. Species of *Madathamugadia* are found in lizards in the Palaearctic and Afrotropics [59–61].

### Molecular results

**Fig. 3:**
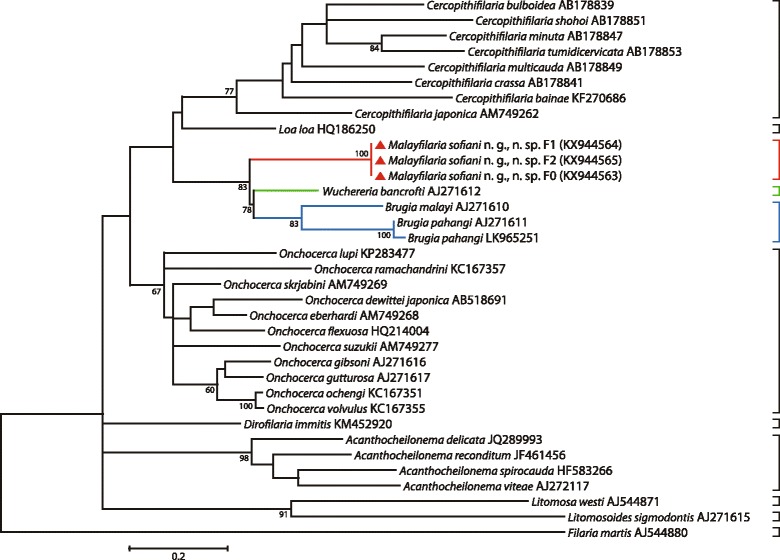
Taxonomic position of *Malayfilaria sofiani* n. g., n. sp., inferred using the maximum-likelihood method, based on *cox*1 nucleotide sequences. The tree was based on the Tamura-Nei model, which was selected as the best model (MEGA6) with 500 replicates. The percentage of replicate trees in which the associated taxa clustered together is shown next to the branches. Values > 50% are shown. The tree is drawn to scale, with branch lengths corresponding to the number of substitutions per site. There were a total of 569 positions in the final dataset. The scale-bar below the diagram indicates the number of changes inferred as having occurred along each branch. The red triangles indicate the sequences generated in this study

**Fig. 4:**
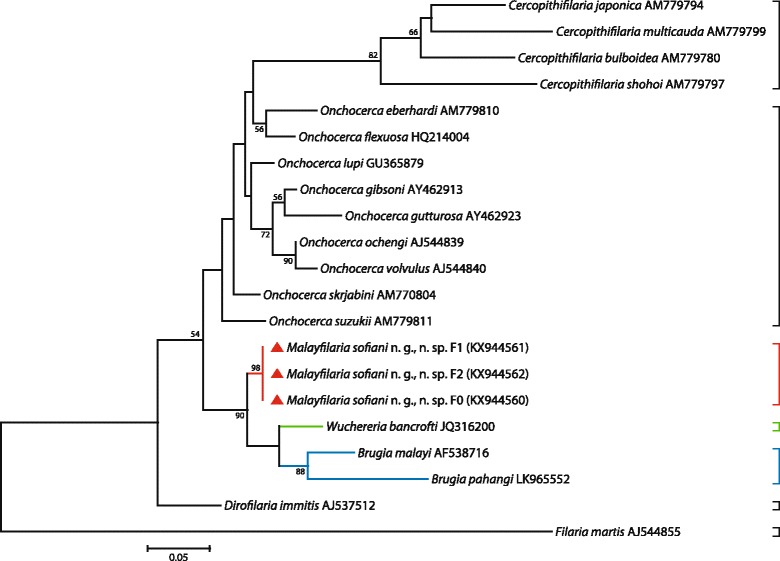
Taxonomic position of *Malayfilaria sofiani* n. g., n. sp., inferred using the maximum-likelihood method, based on 12S rDNA nucleotide sequences. The tree was based on the Tamura-Nei model, which was selected as the best model (MEGA6), with 500 bootstrap replicates. Values > 50% are shown. Gblocks (version 0.91b, 2002) was used to eliminate poorly aligned positions and divergent regions of the alignment, making the data more suitable for phylogenetic analysis [70]. There were 319 positions in the final dataset. The scale-bar below the diagram indicates the number of changes inferred as having occurred along each branch. The red triangles indicate the sequences generated in this study

**Fig. 5:**
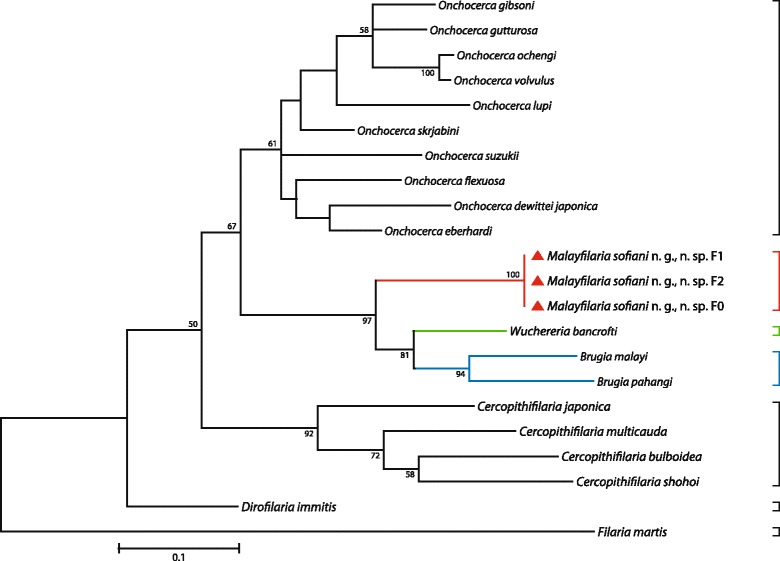
Taxonomic position of *Malayfilaria sofiani* n. g., n. sp., inferred using the maximum-likelihood method, based on combined *cox*1 + 12S rDNA nucleotide sequences. The tree was based on the TN93 + G model, which offered the best fit for the concatenation of the two genetic regions, *cox*1 and 12S rRNA (MEGA6). Values > 50% are shown. There were 888 positions in the final dataset. The scale-bar below the diagram indicates the number of changes inferred as having occurred along each branch. The red triangles indicate the sequences generated in this study

**Fig. 6:**
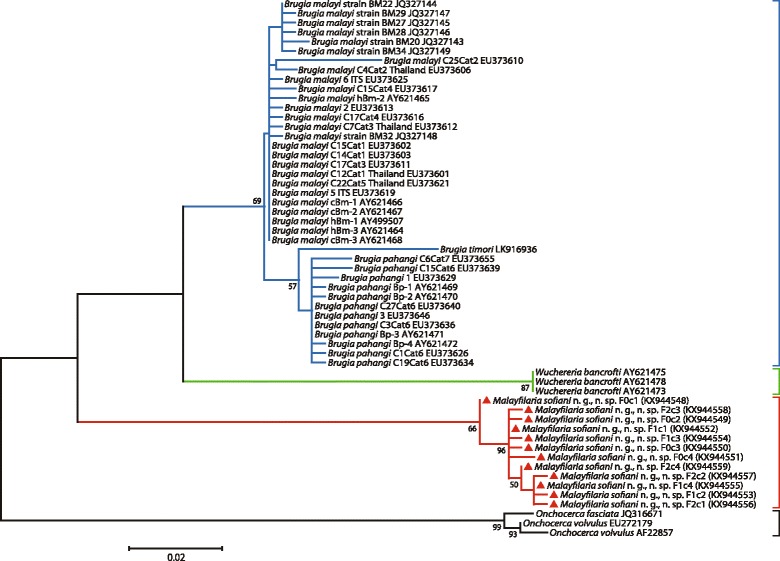
Taxonomic position of *Malayfilaria sofiani* n. g., n. sp., inferred using the maximum-likelihood method, based on ITS1 nucleotide sequences. The tree was based on the Tamura 3 model, with 1,000 bootstrap replicates. Values > 50% are shown. Gblocks (version 0.91b, 2002) was used to eliminate poorly aligned positions and divergent regions of the alignment, making the data more suitable for phylogenetic analysis [70]. There are 495 positions in the final dataset. The scale-bar below the diagram indicates the number of changes inferred as having occurred along each branch. The red triangles indicate the sequences generated in this study

To elucidate the molecular characteristics of *M. sofiani* n. g., n. sp., we compared the nucleotide sequences of the *cox*1 and 12S rRNA genes and the ITS1 region with those of related filarial parasites that were available in the GenBank database. As shown in Additional file 2: Table S2, the K2P distance between sequences of the *cox*1 gene of *M. sofiani* n. g., n. sp. and other known species was 11.8% for *W. bancrofti*, 13.8% for *B. malayi* and 14.1% for *B. pahangi*. Phylogenetic ML trees based on the *cox*1 and 12S rRNA genes placed *M. sofiani* n. g., n. sp. as sister species to the clade formed by *W. bancrofti*, *B. malayi* and *B. pahangi*, indicating that the new species is closer to *W. bancrofti* than to *Brugia* spp. (Figs. 3, 4 and 5). These findings are supported by the tree inferred from the ITS1 region (Fig. 6). The NJ trees for the *cox*1 and 12S rRNA genes and the ITS1 region yielded results similar to the ML trees (see Additional file 3: Figure S1; Additional file 4: Figure S2; and Additional file 5: Figure S3).

The K2P distance between the sequences of the *cox*1 gene of *M. sofiani* n. g., n. sp. and *W. bancrofti* was larger than that between *W. bancrofti* and *B. malayi* (10.6%). The genetic distances between *M. sofiani* n. g., n. sp. and other related species were 13.7% for *B.* (*Breinlia*) *jittapalapongi* Veciana, Bain, Morand, Chaisiri, Douangboupha, Miquel & Ribas, 2015 [55], 13.0–16.9% for *Onchocerca* spp., 17.1–21.5% for *Mansonella* spp. and 15.8–21.5% for *Cercopithifilaria* spp. in the Onchocercinae; 13.2–19.9% for *Loa*, *Dirofilaria* Railliet & Henry, 1910, *Foleyella* and *Pelecitus* in the Dirofilariinae and 13.5–19.4% for *Cardiofilaria*, *Madathamugadia* and *Rumenfilaria* in the Splendidofilariinae. Based on these morphological and molecular comparisons, as well as host data and predilection sites, we conclude that the new species is well distinct from previously described genera and we, thus, propose the new genus *Malayfilaria* in order to accommodate it.

## Discussion

Based on its morphological and molecular characters, *M. sofiani* n. g., n. sp. is most closely related to *Wuchereria* spp. and *Brugia* spp. in the Onchocercinae. To date, eight species belonging to these genera have been recorded from various hosts in the Indo-Malayan region. *Wuchereria bancrofti*, parasitic in humans, occurs throughout the tropics and in some subtropical areas [9], while *W. kalimantani* was first described in silvered leaf monkeys, *Trachypithecus cristatus* (Raffles, 1821) (syn. *Presbytis cristatus* Eschscholtz, 1921), in Kalimantan, Indonesia [44]. Among the ten species belonging to the genus *Brugia* Buckley, 1958, three, *B. malayi*, *B. pahangi* and *B. tupaiae*, have been discovered in humans, monkeys, carnivores and treeshrews in Malaysia [6, 7, 31]. In Sri Lanka, *Brugia buckleyi* Dissanaike & Paramananthan, 1961 has been collected from a Ceylon hare, *Lepus nigricollis singhala* Wroughton, 1915 [49], and *B. ceylonensis* Jayewardene, 1962 has been collected from dogs [50]. In addition, *B. timori* has been found in humans in Indonesia [8].

As detailed above, *M. sofiani* n. g., n. sp. differs from all of these in having an extremely long glandular oesophagus, annulation in the midbody region and small lappets at the terminal end of the tail. In contrast, the pre-oesophageal cuticular ring is a character shared between the three genera. However, despite being mentioned in the description of *Wuchereria* sp. from a monkey [6] and the inclusion of this character in the generic diagnosis of *Brugia* by Buckley [7], detailed descriptions of the pre-oesophageal cuticular ring are so far available for *W. bancrofti* [6, 45, 46], *B. malayi* [47] and *B. patei* [48] only. In this study, we provide metrical data for the pre-oesophageal cuticular ring in *B. pahangi* for the first time.

Among the Onchocercidae, *Acanthocheilonema* spp. also have a salient pre-oesophageal cuticular ring and a stout glandular oesophagus [62, 63], while *Cercopithifilaria* spp. have a small pre-oesophageal cuticular ring and no glandular oesophagus [62]. Chabaud & Bain [64] suggested that *Cercopithifilaria* was derived from the *Acanthocheilonema* lineage, and a stout glandular oesophagus is considered to be one of the ancestral characteristics in *Onchocerca* spp. [65]. Based on this, we infer that the new species has ancestral morphological characteristics when compared with *Wuchereria* spp. and *Brugia* spp.

In the present study, we carefully extracted specimens of *M. sofiani* n. g., n. sp. from the tissues of treeshrews under a stereomicroscope and placed them in phosphate-buffered saline (pH 7.4) in Petri dishes. Regardless of whether or not the worms were fixed in alcohol, we usually observed ornamentation in the midbody region of both sexes of the filarioid (Figs. 1d, 1n, 2c, 2e). Based on the definition provided in the CIH Keys to the Nematode Parasites of Vertebrates [66], we diagnosed this ornamentation as annules. To the best of our knowledge, the presence of annules is a specific characteristic of the new filarioid described here, although various cuticular ornamentations such as transverse ridges, longitudinal ridges and bosses have been identified previously in filarial parasites [2, 46, 67].

Previous phylogenetic trees based on 12S rDNA and *cox*1 sequences [21, 22], as well as 16S rDNA sequences of the endosymbiont bacteria *Wolbachia* in the host filarioids [21, 24], have shown *W. bancrofti* to be close to *Brugia* spp. On the basis of multi-locus sequence analyses, Lefoulon et al. [26] separated taxa of the Onchocercidae into five clades; in one of these, ONC5, *W. bancrofti*, *B. pahangi*, *B. timori* and *B. malayi* formed a monophyletic group. Tree topologies based on the *cox*1 and 12S rRNA genes and the ITS1 region obtained in the present study, placed *M. sofiani* n. g., n. sp. as a sister taxon to the *Wuchereria*-*Brugia* clade, represented by *W. bancrofti* on the one hand and *B. malayi* and *B. pahangi* on the other hand*.* Based on the K2P distance between *cox*1 gene sequences, the new species is closest to *W. bancrofti*.

A total of 81 common treeshrews from various areas in Peninsular Malaysia were examined for *M. sofiani* n. g., n. sp. However, the parasite was present in five host individuals only, all of them captured in Jeram Pasu, Kelantan, a locality on the east coast of Peninsular Malaysia. We conclude that the prevalence of *M. sofiani* n. g., n. sp. is very low and that it parasitises common treeshrews inhabiting a limited geographical area.

Insects belonging to the family Culicidae are known to act as vectors for *W. bancrofti* and *Brugia* spp., as well as species of *Aproctella* and *Foleyella* [1]. By contrast, *M*. (*M*.) *ozzardi* (Manson, 1897) is transmitted by biting midges (Ceratopogonidae) or black flies (Simuliidae), *Loa loa* (Cobbold, 1864) by horse flies (Tabanidae), *P. fulicaeatrae* (Diesing, 1861) by lice (Menoponidae) and *M. hiepei* Hering-Hagenbeck, Boomker, Petit, Killick-Kendrick & Bain, 2000 by sand flies (Psychodidae) [1, 26, 59, 68]. Therefore, although we did not find any potential vectors for *M. sofiani* n. g., n. sp. in the present study, we speculate that the new species is also transmitted by haematophagous arthropods.

*Wuchereria bancrofti* and *W. kalimantani* parasitise humans and monkeys, while *Brugia* spp. parasitise a wide range of hosts, including primates (humans and monkeys), lagomorphs, carnivores and treeshrews [6, 8, 31, 48–53]. Thus, there are no close relationships between the host species of the three filarial genera, *Malayfilaria* n. g., *Wuchereria* and *Brugia*. Phylogenetically, treeshrews belong to the order Scandentia, which is considered to be more closely related to Primates than to Rodentia and Lagomorpha [30]. The common ancestor diverged into Scandentia, Dermoptera and Primates during the Cretaceous (approximately 90 million years ago), and the genus *Tupaia* Raffles, 1821 arose at the end of the Miocene (approximately 10 million years ago) [69]. Roberts et al. [28] suggested that Miocene tectonic events, volcanism and geographical instability drove treeshrew diversification in Southeast Asia; while according to Bain [27], the radiation of nascent filarial lineages may have occurred with the expansion of mammals between the Paleocene and the Pleistocene (66 to 2.5 million years ago).

With regard to diversification of filariae in the *Wuchereria-Brugia* clade, Morales-Hojas [23] suggested that co-speciation between the hosts and parasites was the most plausible driving factor, because some species of *Brugia* and *Wuchereria* parasitise humans and monkeys. However, he emphasised that more information on the phylogenetic relationships between the species within this clade was required to discuss their evolution. Since *M. sofiani* n. g., n. sp. appears to display more ancestral morphological characteristics than either *Wuchereria* spp. or *Brugia* spp., we speculate that the ancestral lineage of the *Wuchereria-Brugia* clade may have arisen in Scandentia and transferred to Primates, Carnivora, Rodentia, Lagomorpha and other mammals *via* its vectors (host switching) and subsequently diversified into *Wuchereria* spp. and *Brugia* spp., rather than evolving *via* host-parasite co-speciation.

## Conclusions

Here, we describe *M. sofiani* n. g., n. sp., found in the tissues surrounding the lymph nodes of the neck of common treeshrews (*T. glis*) in Peninsular Malaysia. Adults of the new species differ from *Wuchereria* spp. and *Brugia* spp. in having a long glandular oesophagus and annules in the midbody region. Molecular analyses based on the *cox*1 gene indicate that *M. sofiani* n. g., n. sp. differs from *W. bancrofti* by 11.8% and from *B. malayi* by 13.8%. Based on both morphological and molecular characteristics, we conclude that *M. sofiani* n. g., n. sp. is close to *W. bancrofti* in the *Wuchereria*-*Brugia* clade.

## Additional files

Additional file 1: Table S1. Comparison of measurements of *Malayfilaria sofiani* n. g., n. sp. from common treeshrews in Malaysia with other filarial species recorded from treeshrews or hosts in the same geographical region. (XLSX 18 kb)

Additional file 2: Table S2. The Kimura 2-parameter (K2P) distance between the sequences of the *cox*1 gene of *Malayfilaria sofiani* n. g., n. sp. and other known species. (XLSX 14 kb)

Additional file 3: Figure S1. Taxonomic position of *Malayfilaria sofiani* n. g., n. sp., inferred using the neighbour-joining method, based on *cox*1 nucleotide sequences. The tree was based on the Kimura 2-parameter model with 10,000 bootstrap replicates (MEGA6). Numbers at the nodes are the bootstrap confidence values after 10,000 replicates. The percentage of replicate trees in which the associated taxa clustered together is shown next to the branches. Values > 50% are shown. There were a total of 569 positions in the final dataset. The scale-bar below the diagram indicates the number of changes inferred as having occurred along each branch. Red triangles indicate the sequences generated in this study. (PDF 290 kb)

Additional file 4: Figure S2. Taxonomic position of *Malayfilaria sofiani* n. g., n. sp., inferred using the neighbour-joining method, based on 12S rDNA nucleotide sequences. The tree was based on the Kimura 2-parameter model with 10,000 bootstrap replicates (MEGA6). Gblocks (version 0.91b, 2002) was used to eliminate poorly aligned positions and divergent regions of the alignment [70]. There were 319 positions in the final dataset. The scale-bar indicates the number of changes inferred as having occurred along each branch. Red triangles indicate the sequences generated in this study. (PDF 297 kb)

Additional file 5: Figure S3. Taxonomic position of *Malayfilaria sofiani* n. g., n. sp., inferred using the neighbour-joining method, based on ITS1 nucleotide sequences. The tree was based on the Kimura 2-parameter model with 1,000 bootstrap replicates (MEGA6). Gblocks was used to eliminate poorly aligned positions and divergent regions of a DNA alignment [70]. There are 495 positions in the final dataset. The scale-bar indicates the number of changes inferred as having occurred along each branch. Red triangles indicate the sequences generated in this study. (PDF 170 kb)

## Abbreviations

*cox*1: cytochrome *c* oxidase subunit 1
ITS1: internal transcribed spacer 1
K2P: Kimura 2-parameter
NJ: neighbour-joining
ML: maximum-likelihood
MNHN: Muséum National d’Histoire Naturelle
CIH: Commonwealth Institute of Helminthology
