# Supplementary figures and images for: Morphological and molecular characteristics of Malayfilaria sofiani Uni, Mat Udin & Takaoka n. g., n. sp. (Nematoda: Filarioidea) from the common treeshrew Tupaia glis Diard & Duvaucel (Mammalia: Scandentia) in Peninsular Malaysia
Source: Parasit Vectors. 2017 Apr 20;10:194. doi: 10.1186/s13071-017-2105-9 (PMC5397817; doi:10.1186/s13071-017-2105-9)

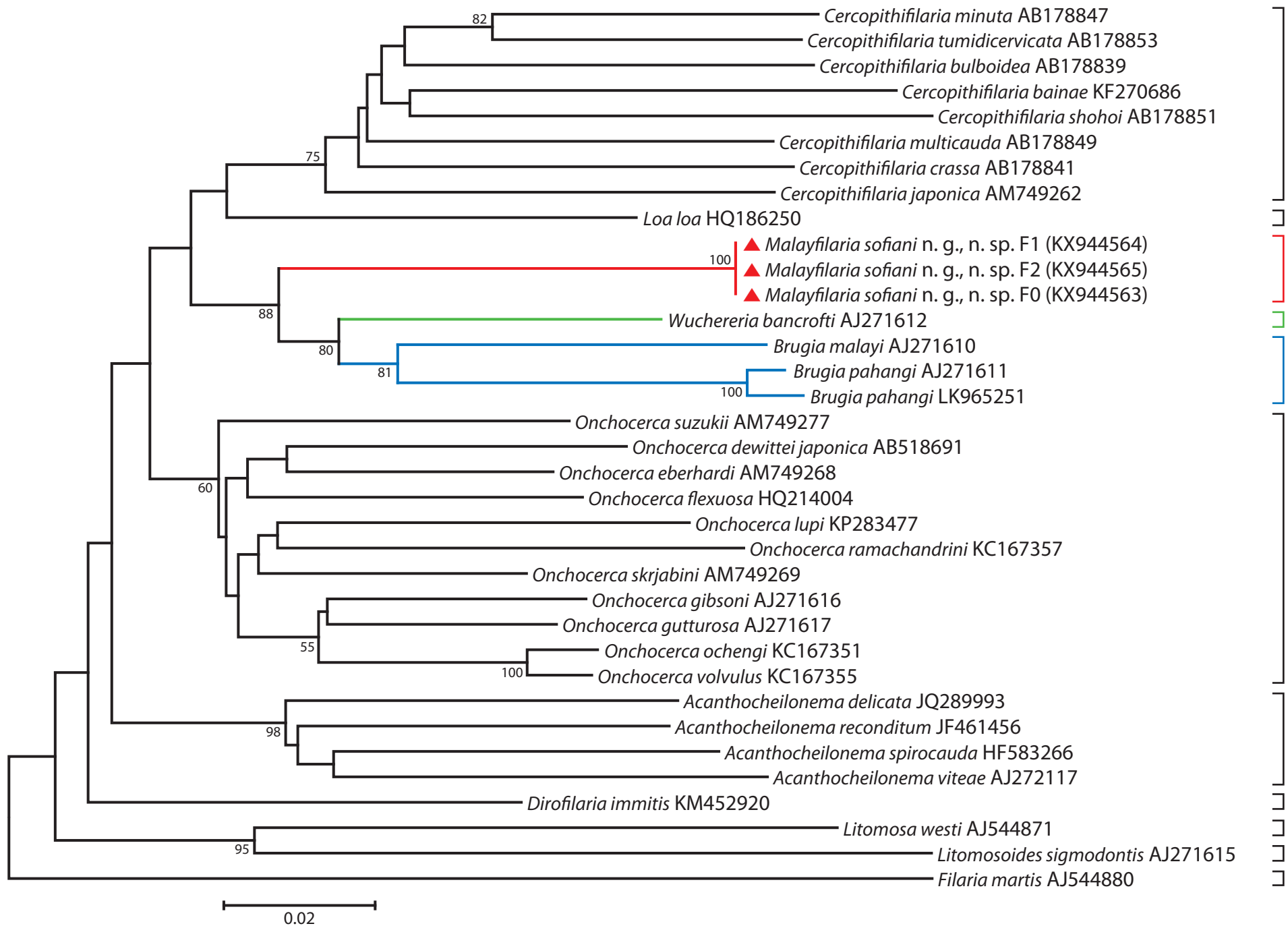

Fig. S1

Supplement: Supplementary file 3 — Taxonomic position of Malayfilaria sofiani n. g., n. sp., inferred using the neighbour-joining method, based on cox1 nucleotide sequences. The tree was based on the Kimura 2-parameter model with 10,000 bootstrap replicates (MEGA6). Numbers at the nodes are the bootstrap confidence values after 10,000 replicates. The percentage of replicate trees in which the associated taxa clustered together is shown next to the branches. Values > 50% are shown. There were a total of 569 positions in the final dataset. The scale-bar below the diagram indicates the number of changes inferred as having occurred along each branch. Red triangles indicate the sequences generated in this study. (PDF 290 kb) [file 13071_2017_2105_MOESM3_ESM.pdf]

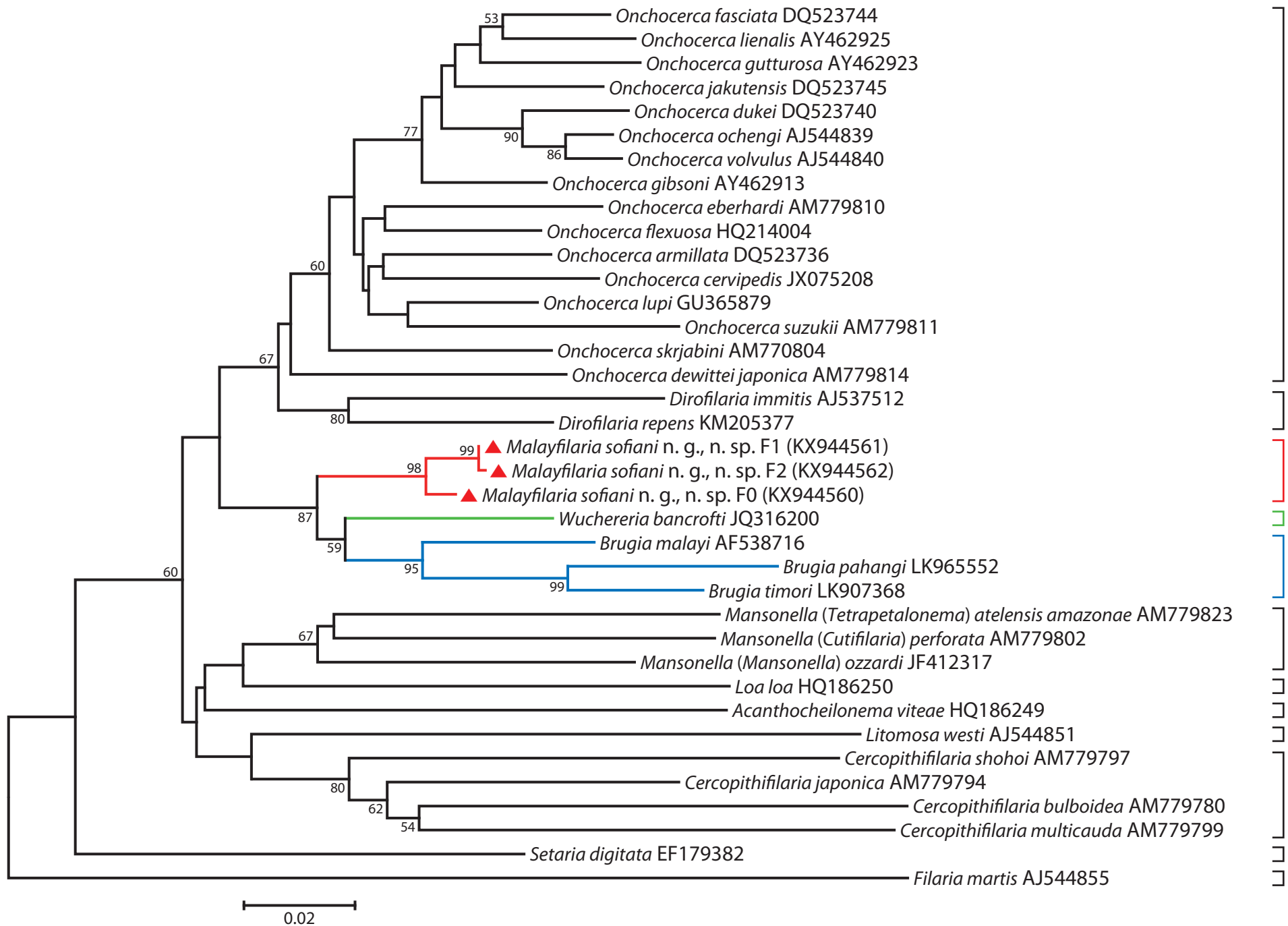

Fig. S2

Supplement: Supplementary file 4 — Taxonomic position of Malayfilaria sofiani n. g., n. sp., inferred using the neighbour-joining method, based on 12S rDNA nucleotide sequences. The tree was based on the Kimura 2-parameter model with 10,000 bootstrap replicates (MEGA6). Gblocks (version 0.91b, 2002) was used to eliminate poorly aligned positions and divergent regions of the alignment [70]. There were 319 positions in the final dataset. The scale-bar indicates the number of changes inferred as having occurred along each branch. Red triangles indicate the sequences generated in this study. (PDF 297 kb) [file 13071_2017_2105_MOESM4_ESM.pdf]

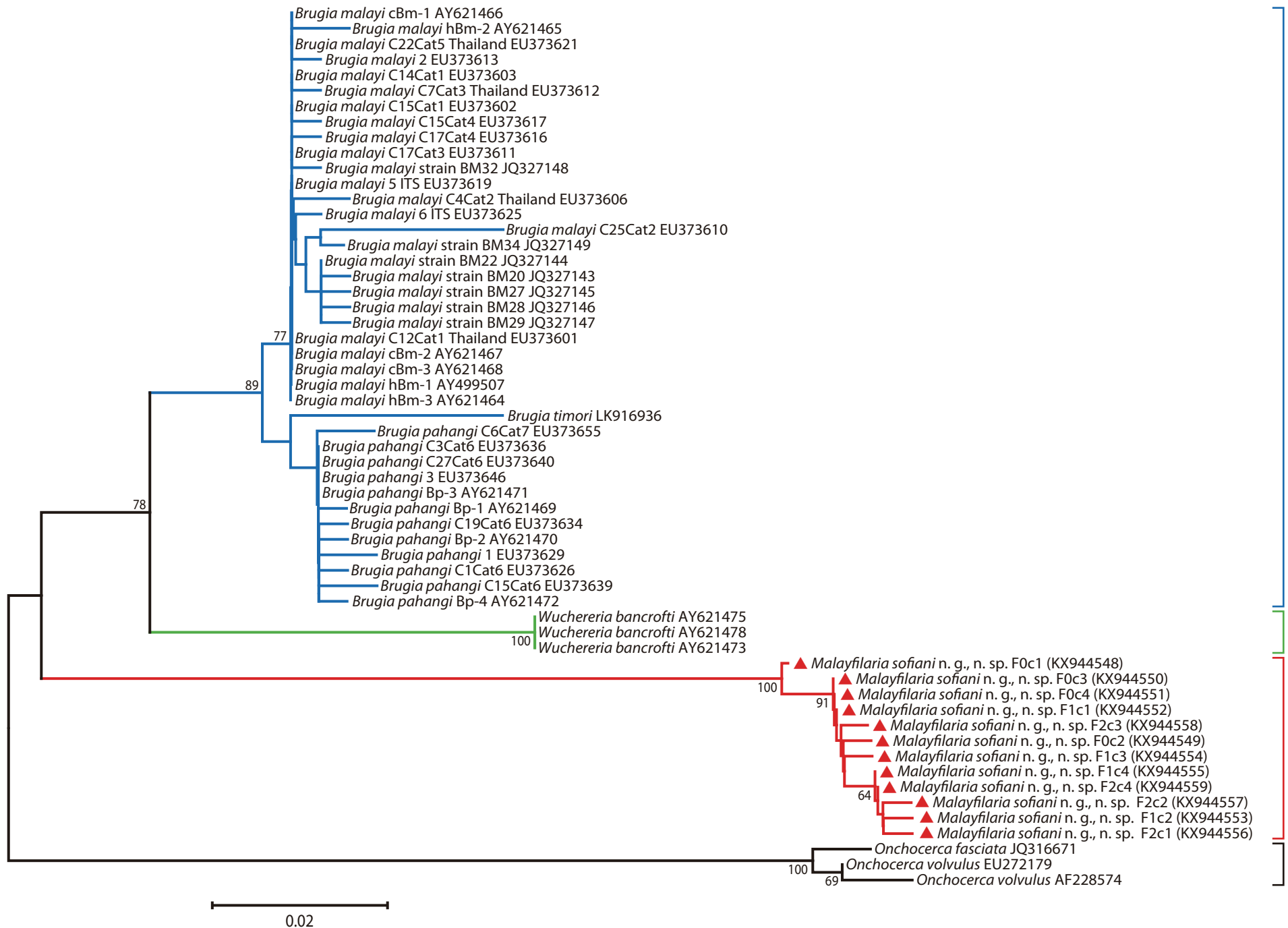

Fig. S3

Supplement: Supplementary file 5 — Taxonomic position of Malayfilaria sofiani n. g., n. sp., inferred using the neighbour-joining method, based on ITS1 nucleotide sequences. The tree was based on the Kimura 2-parameter model with 1,000 bootstrap replicates (MEGA6). Gblocks was used to eliminate poorly aligned positions and divergent regions of a DNA alignment [70]. There are 495 positions in the final dataset. The scale-bar indicates the number of changes inferred as having occurred along each branch. Red triangles indicate the sequences generated in this study. (PDF 170 kb) [file 13071_2017_2105_MOESM5_ESM.pdf]
